# Mucinous adenocarcinoma of renal pelvis in a young male: a diagnostic challenge

**DOI:** 10.3205/000287

**Published:** 2020-11-25

**Authors:** Pooja Gupta, Deepti Agarwal, Sharma Shruti, Mithlesh Chandra

**Affiliations:** 1 Department of Pathology, National Institute of Pathology, New Delhi, India; 2 Department of Pathology, Pathology Consultancy Services, Noida, India

**Keywords:** mucinous adenocarcinoma, hydronephrosis, urothelial tumor, urolithiasis

## Abstract

Primary mucinous adenocarcinoma of the renal pelvis is an extremely rare tumor with only a handful of cases reported to date. Clinical and radiological features are not specific, and hence, histopathological examination holds the key for definitive diagnosis. This tumor has mainly been described in the elderly population, with less than five cases reported in individuals aged <35 years. Here, we report a case of primary mucinous adenocarcinoma of the renal pelvis in a young male. A 31-year-old male presented with a history of right-sided flank pain for the past year. On examination, he had right-sided costovertebral tenderness. Computed tomography (CT) scan revealed the presence of a hyperdense mass lesion in the right renal pelvis with severe hydronephrosis and cortical thinning. Because of the non-functioning status, right nephrectomy was performed. To our surprise, histopathology showed the presence of mucinous adenocarcinoma of the renal pelvis with carcinoma in situ of the ureter. This case describes a rare presentation of primary mucinous adenocarcinoma of the renal pelvis, and highlights the importance of histopathological examination in reaching the correct diagnosis.

## Zusammenfassung

Das primäre muzinöse Adenokarzinom des Nierenbeckens ist ein extrem seltener Tumor, zu dem bisher nur eine Handvoll Fälle publiziert wurden. Da klinische und radiologische Merkmale nicht spezifisch sind, ist die histopathologische Untersuchung entscheidend für die definitive Diagnose. Dieser Tumor wurde hauptsächlich bei älteren Menschen beschrieben, mit weniger als fünf Fällen bei Personen im Alter von <35 Jahren. Hier berichten wir über einen Fall von primärem muzinösem Adenokarzinom des Nierenbeckens bei einem jungen Patienten. Ein 31-jähriger Mann stellte sich mit einer einjährigen Vorgeschichte von rechtsseitigen Flankenschmerzen vor. Bei der Untersuchung hatte er eine rechtsseitige costovertebrale Druckempfindlichkeit. Bei einer Computertomographie (CT) zeigte sich eine hyperdense Massenläsion im rechten Nierenbecken mit schwerer Hydronephrose und kortikaler Ausdünnung. Aufgrund des funktionslosen Status der Niere wurde eine rechtsseitige Nephrektomie durchgeführt. Zu unserer Überraschung zeigte die Histopathologie das Vorhandensein eines muzinösen Adenokarzinoms des Nierenbeckens mit Karzinom in situ des Harnleiters. Dieser Fall beschreibt eine seltene Darstellung des primären muzinösen Adenokarzinoms des Nierenbeckens und unterstreicht die Bedeutung der histopathologischen Untersuchung für die Feststellung der richtigen Diagnose.

## Introduction

Primary adenocarcinoma accounts for about 0.5–2.0% of all malignant tumors of the urinary tract [1]. The most common sites for this tumor are urinary bladder, followed by ureter and renal pelvis. Primary mucinous adenocarcinoma of the renal pelvis is an extremely rare tumor, which has only been reported through a handful of cases to date. Herein, we report a case of a 31-year-old-male, who was suspected to have renal stone disease radiologically, but diagnosed to be having mucinous adenocarcinoma of the renal pelvis with carcinoma in situ of ureter based on the histopathological profile.

## Case description

**Figure 1:**
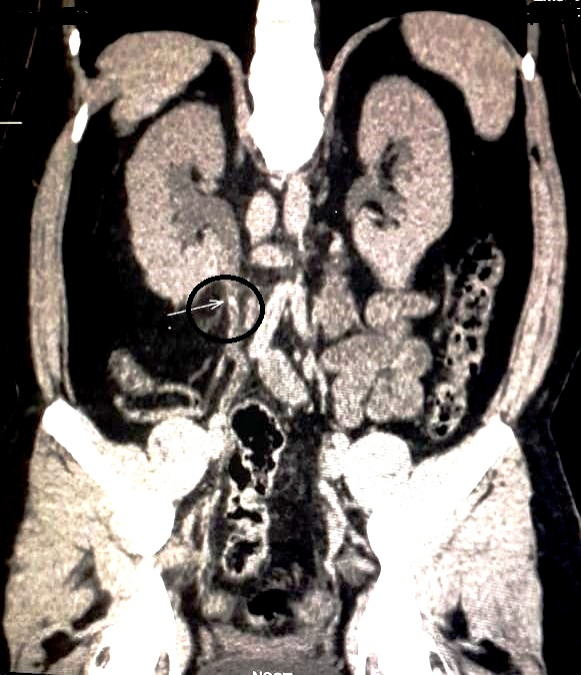
Computed tomography (CT) of the abdomen showing a mass lesion in the right renal pelvis

**Figure 2:**
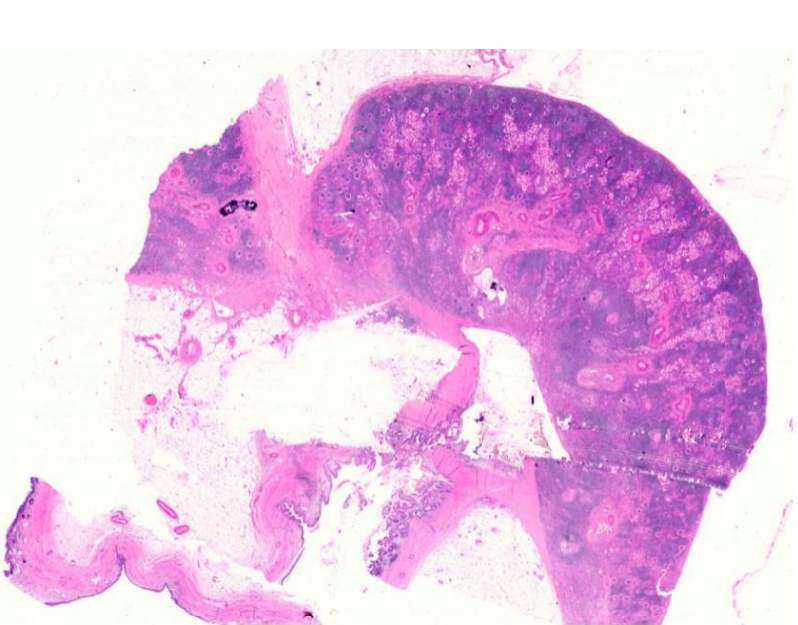
Scanner view showing mucinous adenocarcinoma involving the renal pelvis (H&E, 20X)

**Figure 3:**
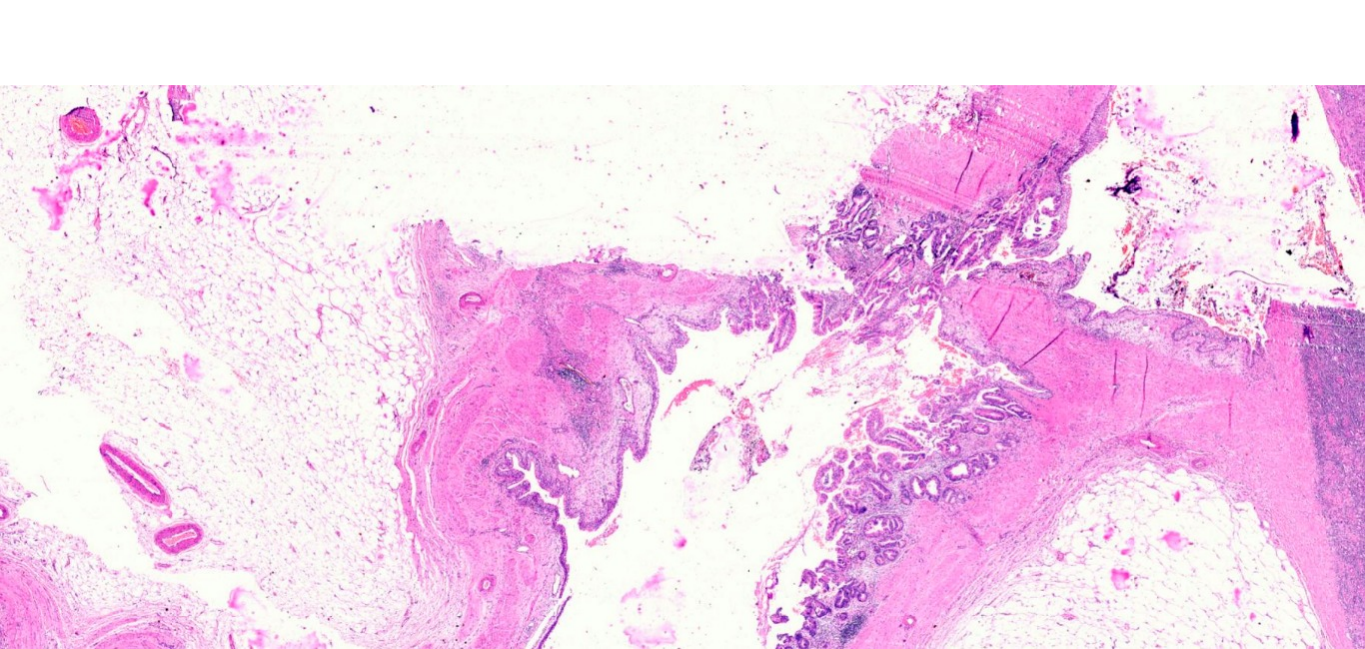
Mucinous adenocarcinoma involving the renal pelvis (H&E, 200x)

**Figure 4:**
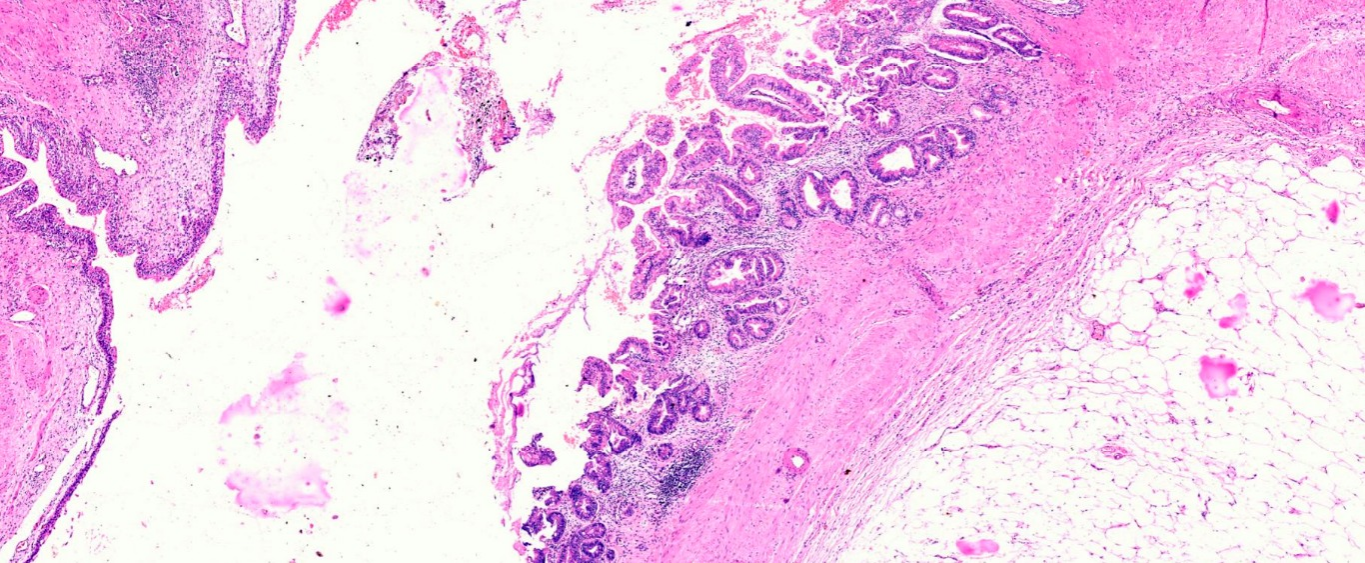
Tumor infiltrating the lamina propria and superficial layers of muscularis propria (H&E stain, 400X)

**Figure 5:**
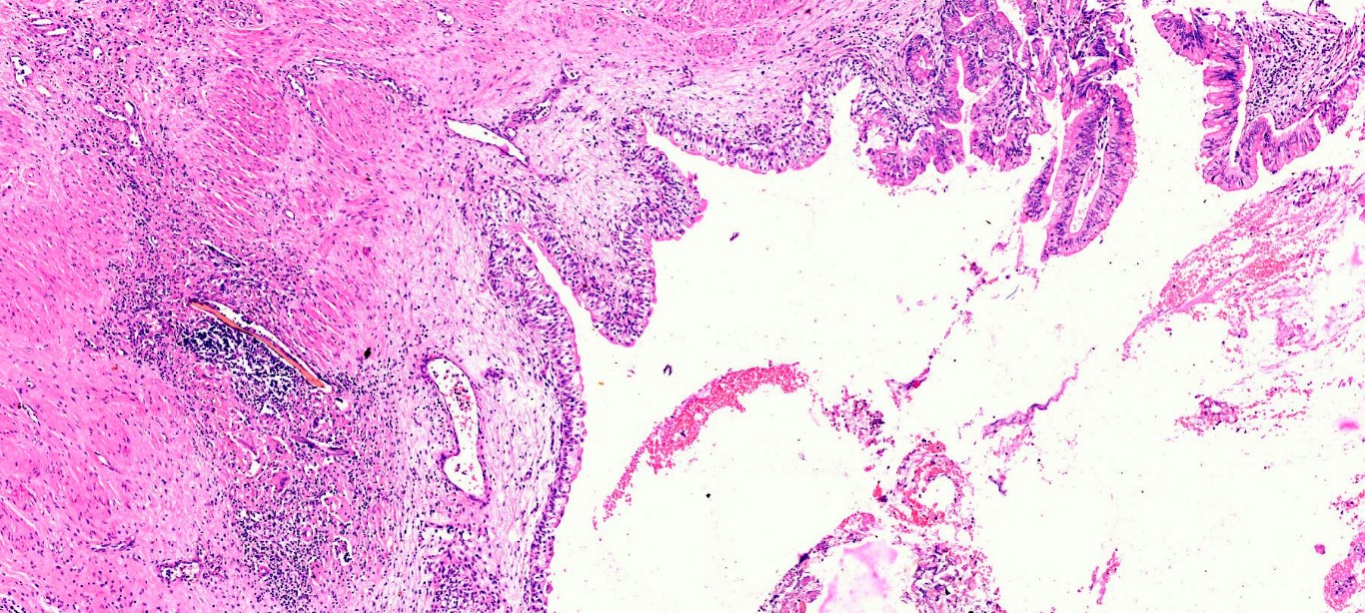
Showing transition of normal transitional epithelial lining to malignant columnar lining (H&E stain, 400X)

**Figure 6:**
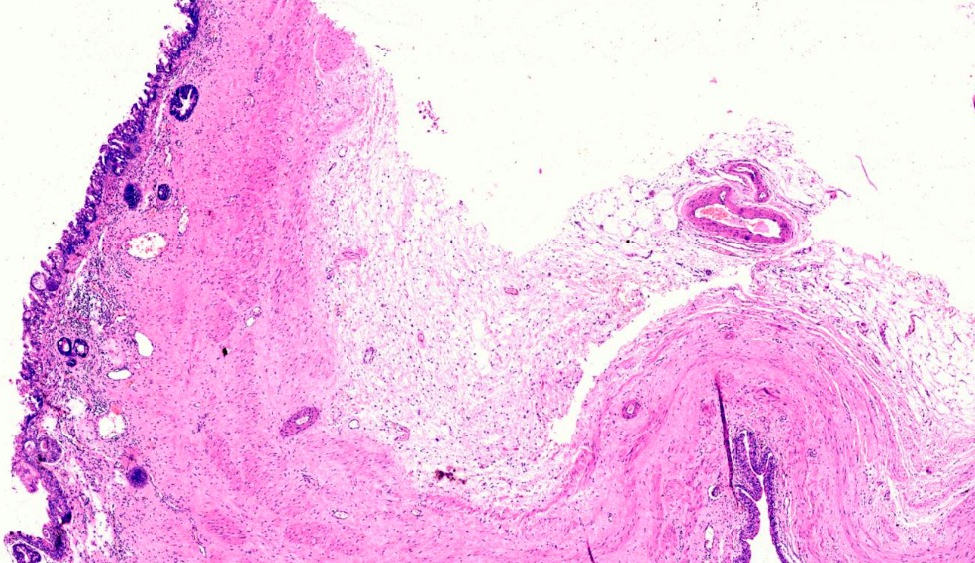
Adenocarcinoma in situ involving the ureter (H&E stain, 200X)

A 31-year-old male of Asian-Indian origin presented to the outpatient department with a one-year history of dull aching right-sided flank pain. There were associated symptoms of abdominal distension and difficulty in voiding urine for the same duration. There was no history of polyuria, graveluria, hematuria or dysuria. On examination, right costovertebral angle tenderness was noted. General and systemic examinations were unremarkable. No lymphadenopathy was noted. On investigation, complete blood count and liver and renal function tests were within normal limits. Urine microscopy showed increased pus cells (20–30/high power field); there were no red blood cells, casts, crystals or malignant cells. Ultrasonography of kidney, ureter and bladder region revealed right-sided hydronephrosis. Computed tomography (CT) scan revealed a hyperdense mass of 2.3x4.6 mm in the right renal pelvis with severe hydronephrosis and cortical thinning (Figure 1 (Fig. 1)). Renal dynamic scan revealed a poorly functioning right-sided kidney. Considering the poor functional status, right-sided nephrectomy was performed and the specimen was sent for histopathological examination. On gross examination, the kidney measured 11x8x5 cm with a cut surface showing dilated pelvicalyceal system filled with slimy watery fluid; the renal pelvis was thickened with a greyish-white cut surface. Multiple stones were noted in the pelvis and kidney parenchyma. Microscopic examination revealed features of chronic pyelonephritis; the adjacent renal pelvis showed pseudostratified columnar epithelial metaplasia with presence of tumor cells arranged in the form of glands and suspended in mucin pools. The tumor infiltrated lamina propria and superficial layers of the muscle coat of the renal pelvis (Figure 2 (Fig. 2), Figure 3 (Fig. 3), Figure 4 (Fig. 4), Figure 5 (Fig. 5)). Adenocarcinoma in situ was noted in the adjacent ureter (Figure 6 (Fig. 6)). Pathological TNM staging was pT_2a_N_x_M_x_.

The resected margins were free from tumor. A diagnosis of mucinous adenocarcinoma of the right renal pelvis with carcinoma in situ of adjacent ureter was made. The patient did not receive adjuvant radiotherapy or chemotherapy. He has remained disease-free on clinical grounds until six months of postoperative follow-up.

## Discussion

Transitional cell carcinomas (TCC) are the most common type of urothelial malignancy. On the other hand, primary adenocarcinoma of the urothelial tract is uncommon, accounting for less than 1% of all the malignant tumors in this region [1], [2]. The peak incidence of primary adenocarcinoma of the urothelial tract has been reported in the sixth and seventh decades of life. Its occurrence in the younger population is extremely rare, with only three cases reported in patients aged <35 years [3], [4], [5]. The histogenetic profile of this rare entity is not clearly known and is postulated to be related to chronic irritation of the lining epithelium due to urolithiasis, pyelonephritis, and hydronephrosis. This may result in glandular metaplasia, eventually developing into mucinous adenocarcinoma [6]. The clinical and radiological presentation is varied and often overlaps with renal stone disease, thus accounting for the difficulty in preoperative diagnosis. Often, patients present late with vague symptoms like flank pain and abdominal distension with or without hematuria when the tumor is already at an advanced stage. It is important to exclude metastasis from primary sites such as urachus, prostate, colon, female genital tract, appendix, stomach, and breast before making the diagnosis of this rare tumor. The closest differential is metastatic urachal adenocarcinoma (Table 1 (Tab. 1)) [7], [8], [9], [10]. Since metastasis from urachal adenocarcinoma may closely mimic adenocarcinoma of the urothelial region, a comparison of histopathological and immunohistochemical features of these two malignancies has been provided in Table 2 (Tab. 2) [11]. The typical immunohistochemical profile of adenocarcinoma of the urothelial region comprises of positivity for CK7, CK20, CDX2, EMA, villin, MUC1, MUC2, and MUC3 [12].

Surgery is the treatment of choice for mucinous adenocarcinoma of the renal pelvis, standard therapy being radical nephroureterectomy and bladder cuff excision [13], [14]. Due to the rarity of this tumor and lack of properly representative data, the role of adjuvant chemotherapy or radiotherapy is still not clear. The overall prognosis has been reported as relatively poor in the majority of studies [9], [15].

## Conclusion

To conclude, mucinous adenocarcinoma of the renal pelvis is an extremely rare tumor whose preoperative diagnosis is almost always missed, making histopathology crucial. The case has been reported in view of its rarity and occurrence in a young patient. This report will enrich the current literature on this rare variety of tumors.

## Notes

### Competing interests

The authors declare that they have no competing interests.

### Acknowledgment

We would like to acknowledge Dr. Mayank Gupta (consultant urologist, Neo Hospital, Noida, India) for performing surgical intervention on the patient.

## Figures and Tables

**Table 1:**
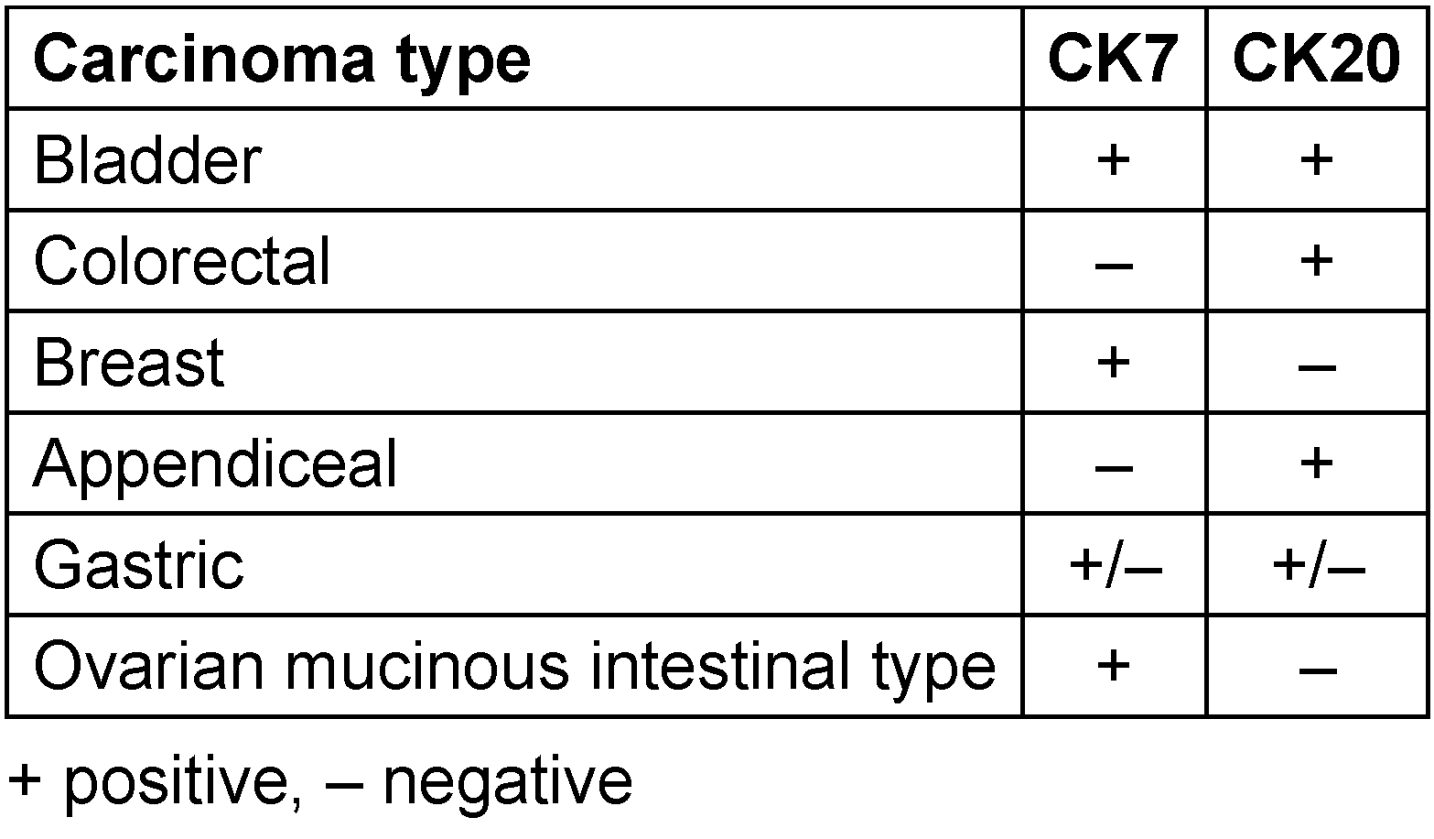
Immunophenotypic distribution of markers for various differentials of adenocarcinoma of the renal pelvis

**Table 2:**
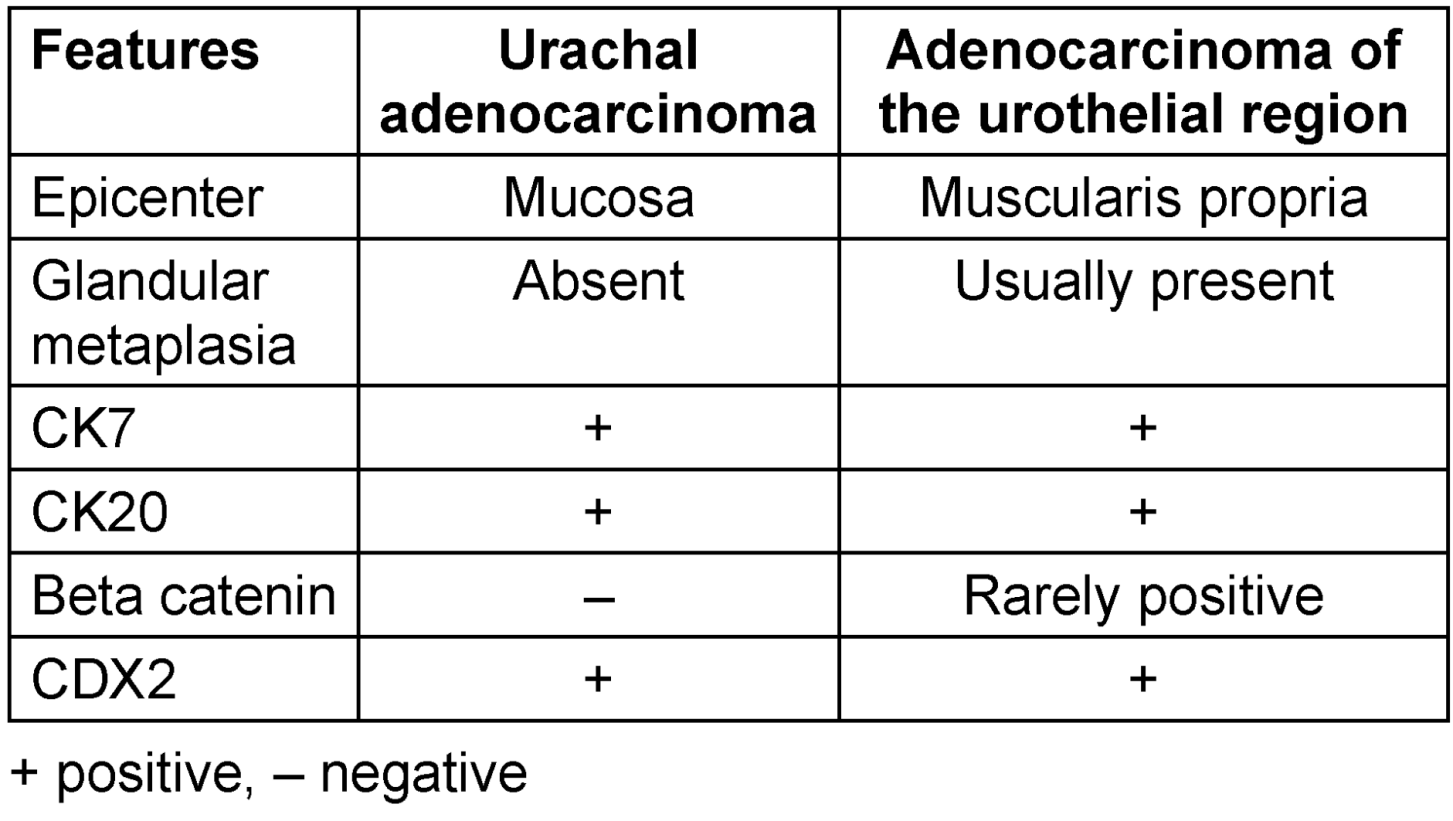
Histopathological and immunohistochemical differences between urachal adenocarcinoma and adenocarcinoma of the urothelial region
